# CRISPR/Cas9 TCR-Edited NKp30 CAR T Cells Exhibit Superior Anti-Tumor Immunity to B7H6-Expressing Leukemia and Melanoma

**DOI:** 10.3390/ijms26178235

**Published:** 2025-08-25

**Authors:** Sedigheh Givi, Benedikt J. Lohnes, Saber Ebrahimi, Sophie Riedel, Sneha Khokhali, Shamsul A. Khan, Maximilian Keller, Catherine Wölfel, Hakim Echchannaoui, Ernesto Bockamp, Maya C. Andre, Hinrich Abken, Matthias Theobald, Udo F. Hartwig

**Affiliations:** 1 Department of Medicine, Hematology & Medical Oncology, University Medical Center of Johannes Gutenberg University, 55101 Mainz, Germany; s_givi@yahoo.com (S.G.); sriedel@students.uni-mainz.de (S.R.); skhokhal@students.uni-mainz.de (S.K.); keller_maximilian@hotmail.de (M.K.); cwoelfel@uni-mainz.de (C.W.); echchann@uni-mainz.de (H.E.); direktor-3med@unimedizin-mainz.de (M.T.); 2 Research Center for Immunotherapy, University Medical Center of Johannes Gutenberg University, 55101 Mainz, Germany; bockamp@uni-mainz.de; 3 Partner Site Frankfurt/Mainz, German Consortium for Translational Cancer Research (DKTK), 55101 Mainz, Germany; 4 Institute for Translational Immunology, University Medical Center of Johannes Gutenberg University, 55101 Mainz, Germany; 5 Department of Hematology/Oncology and General Pediatrics, Children’s University Hospital, University of Tübingen, 72074 Tübingen, Germany; mayacaroline.andre@googlemail.com; 6 Department of Pediatric Intensive Care, Children’s University Hospital, 4031 Basel, Switzerland; 7 Division Genetic Immunotherapy, Leibniz Institute for Immunotherapy, 93053 Regensburg, Germany; hinrich.abken@ukr.de

**Keywords:** immunotherapy, acute myeloid leukemia, melanoma, CAR T-cell therapy, B7H6 expressing tumors, NKp30-based CAR T-cell therapy

## Abstract

Chimeric antigen receptor (CAR) T-cell therapy directed to CD19 and B-cell maturation antigen has revolutionized treatment of B-cell leukemia and lymphoma, and multiple myeloma. However, identifying suitable targets for acute myeloid leukemia (AML) remains challenging due to concurrent expression of potential target antigens on normal hematopoietic stem cells or tissues. As the stress-induced B7H6 molecule is rarely found on normal tissues but expressed on many cancers including AML and melanoma, the NKp30-ligand B7H6 emerges as a promising target for NKp30-based CAR T therapy for these tumors. In this study, we report a comprehensive B7H6 expression analysis on primary AML and melanoma as well as on different tumor cell-lines examined by RT-qPCR and flow cytometry, and efficient anti-tumor reactivity of NKp30-CAR T cells to AML and melanoma. To overcome limitations of autologous CAR T-cell fitness-dependent efficacy and patient-tailored production, we generated CRISPR/Cas9-mediated TCR-knockout (TCR^KO^) NKp30-CAR T cells as an off-the-shelf approach for CAR T therapy. Functional studies comparing NKp30-CD28 CAR or NKp30-CD137 CAR TCR^+^ and TCR^KO^ T lymphocytes revealed superior anti-tumoral immunity of NKp30-CD28 CAR TCR^KO^ T cells to AML and melanoma cell lines in vitro, and effective control of tumor burden in an NSG melanoma-xenograft mouse model. In conclusion, these findings highlight the therapeutic potential of NKp30 CAR TCR^KO^ T cells for adoptive T-cell therapy to B7H6-expressing cancers, including melanoma and AML.

## 1. Introduction

Chimeric antigen receptor (CAR) T-cell therapy has revolutionized conventional cancer therapy regimens. In particular, engineered T cells with a CAR directed to CD19 or B-cell maturation antigen (BCMA) have become integral in the treatment of relapsed/refractory B-cell leukemia and lymphoma, as well as multiple myeloma, stated in [1,2,3].

However, in contrast to remarkable response rates achieved by CAR T therapy for these malignancies, CAR T cells directed to acute myeloid leukemia (AML) revealed only limited success [4]. In addition to obstacles such as genetic heterogeneity of AML with variable antigen expression and different factors contributing to an immunosuppressive bone marrow microenvironment [5], the main limitation of CAR T therapy in AML is the lack of specific antigens that are selectively expressed on leukemic blasts and ideally on leukemic stem cells, but not in their healthy counterparts, such as hematopoietic stem cells (HSCs) [4,6]. So far, potential targets already elaborated in clinical studies comprise the myeloid-lineage specific markers CD123 (IL-13 receptor alpha chain) [7], CD33 [8], and C-type lectin-like molecule 1 (CLL-1) [9], which is also expressed on AML cells that lack CD33. Furthermore, CAR T therapy utilizing NKG2D-based [10] and anti-Lewis-Y (LeY) CAR T cells [11] have been evaluated in clinical phase I studies to target NKG2D ligands and LeY antigen expressing AML blasts. In addition, antigens under preclinical investigation include CD135 (FLT-3 receptor) [12], Folate receptor β [13], CD44v6 [14], B7-H3 [15], CD70 [16], and CD7 [17]. However, none of the antigens studied in clinical trials fulfill the requirements for a tumor-selective, therapeutically suitable antigen due to their potentially therapy-limiting on-target, off-tumor toxicity or limited therapeutic efficacy. Targeting other antigens elicited limited anti-leukemic reactivity despite no or minimal expression on healthy tissue [4]. Among the target candidates examined preclinically so far, only targeting CD70 has shown some premises in a xenograft model without significant off-target activity [4].

Alternatively, the B7 homologous H6 (B7H6) protein, a member of the human-specific B7 family, appears to be a very promising target for CAR T therapy [18,19]. Unlike other B7 members, cell surface-expressed B7H6 is usually absent from healthy tissues under steady state conditions, including HSC, but de novo upregulated as a stress-induced response protein [20] not only on hematological but also on a broad range of solid cancers, including melanoma [18,19,21,22,23]. Recently, bromodomain-containing protein 4 (BRD4), which is overexpressed in AML, was reported to elevate B7H6 expression in AML blasts, and patients with high B7H6 expression showed poor prognosis, further strengthening B7H6 as a therapeutic target [24].

Moreover, B7H6 is the primary ligand recognized by the activating natural cytotoxicity receptor (NCR) NKp30 expressed on natural killer (NK) and NK-T cells and has been shown to play an essential role in NK- and NK-T cell-mediated tumor cell killing [18,22,25].

To validate B7H6 as target for CAR T therapy in AML and melanoma, we first analyzed B7H6 expression in patient-derived AML and melanoma samples at the mRNA transcript and cell-surface level in comparison to well-known AML and melanoma cell lines. B7H6 surface expression was found in the majority of primary AML and all melanoma samples tested. Additionally, we engineered human T cells expressing a second-generation NKp30-based CAR composed of the extracellular domain of NKp30 fused to either CD28 or 4-1BB (CD137) costimulatory and the CD3ζ signal transduction domains. To overcome limitations of autologous CAR T cell fitness-dependent efficacy and individual manufacturing, we applied allogeneic T lymphocytes from healthy donors to deplete TCRαβ expression by the clustered regularly interspaced short palindromic repeat (CRISPR)/CRISPR-associated (Cas) 9 nuclease (CRISPR/Cas-9) editing, followed by engineering of NKp30-CAR redirected T cells. Both NKp30-CD28 CAR TCR^KO^ and NKp30-CD137 CAR TCR^KO^ CAR T cells exerted potent anti-tumoral responses to AML and the melanoma cell line A375 in vitro, while NKp30-CD28 CAR TCR^KO^ T cells elicited superior responses and sustained tumor burden control in vivo in a preclinical NSG xenograft tumor model.

These studies demonstrate that human NKp30 CAR TCR^KO^ T cells elicit potent antitumor immunity, extending previous findings on NKp30-based or anti-B7H6 scFv CAR-redirected murine T cells [26,27], and highlight the therapeutic potential of engineered allogeneic NKp30 CAR T cells for CAR T therapy to B7H6-expressing cancers, including not only solid tumors such as melanoma, but importantly to AML as well.

## 2. Results

### 2.1. Generation of NKp30-Based CARs and Expression in Human TCR^KO^ T Lymphocytes

**Figure 1:**
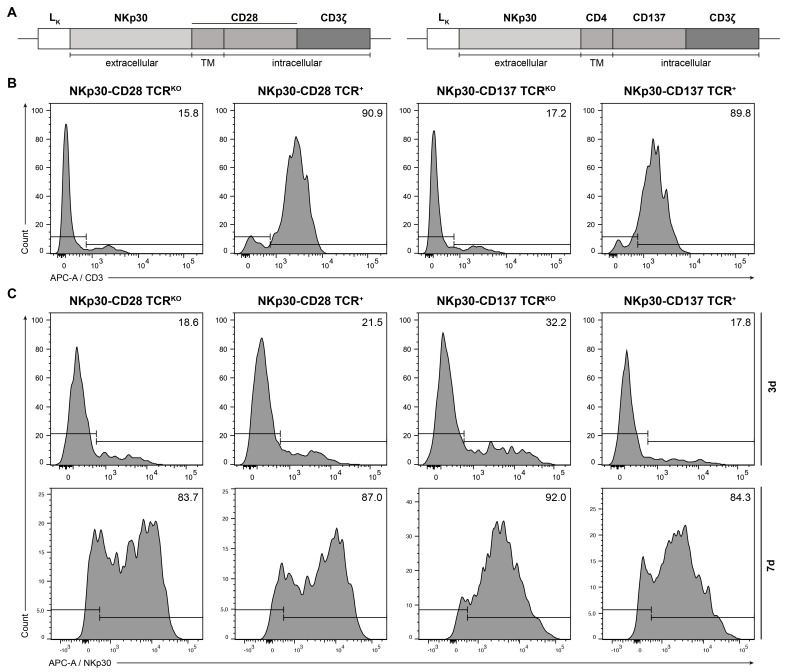
Validation of TCR knockout and CAR expression by flow cytometry. (**A**) Second-generation NKp30-based CAR constructs were generated by fusing the extracellular domain of NKp30 to either a CD28 TMD and ICD (NKp30-CD28) or a CD4 TMD and CD137 ICD (NKp30-CD137), followed by the CD3ζ domain. (**B**) After retroviral transduction of PBMCs, efficacy of CRISPR/Cas9-mediated knockout of the endogenous TCR was analyzed by flow cytometry measuring surface CD3 expression 72 h post transduction. (**C**) Antibiotic selection was applied to enrich CAR-expressing cells. Surface expression of the NKp30-CAR was quantified at multiple time points by flow cytometry using an anti-NKp30 mAb. Fluorescence signals were acquired using a BD FACS Canto II flow cytometer. A representative flow cytometric analysis is shown.

To establish second-generation CARs composed of the ectodomain of human NKp30 and either CD28 or 4-1BB (CD137) costimulatory domains followed by the CD3ζ signaling moiety, we cloned sequences encoding the CD28 transmembrane domain (TMD), the intracellular domain (ICD), and the CD3ζ domain without a spacer element downstream of the extracellular region of NKp30, into a selectable retroviral transfer vector (Figure 1A). Accordingly, a NKp30-based CAR cassette containing a CD4 TMD and CD137 ICD followed by the CD3ζ domain was inserted into the same vector (Figure 1A).

As we explored allogeneic human T lymphocytes to be utilized for CAR T therapy, we next applied CRISPR/Cas9 editing to induce a double-strand break in the TCR α-chain of polyclonally activated TCRαβ T cells upon transfection of a gRNA/Cas9 nucleoprotein complex. Following a 4 hour (h) culture TCR-knockout (KO), T cells were subsequently subjected to retroviral gene transfer to obtain NKp30-CD28 and NKp30-CD137 CAR-redirected TCR^KO^ T cells and their TCR^+^ counterparts for comparative studies (Figure 1). T cells analyzed three days after genetic engineering by flow cytometry (Figure 1B,C) showed 16–17% residual CD3 (Figure 1B) and 18–32% NKp30 CAR expression (Figure 1C), while CAR expressing T cell subsets increased to more than 83% at day seven after puromycin selection (Figure 1C). These data demonstrated that human T cells of healthy donors can be successfully engineered to largely abrogate TCR expression while expressing high levels of CAR.

### 2.2. B7H6 Is Widely Expressed on the Surface of AML and Melanoma

**Figure 2:**
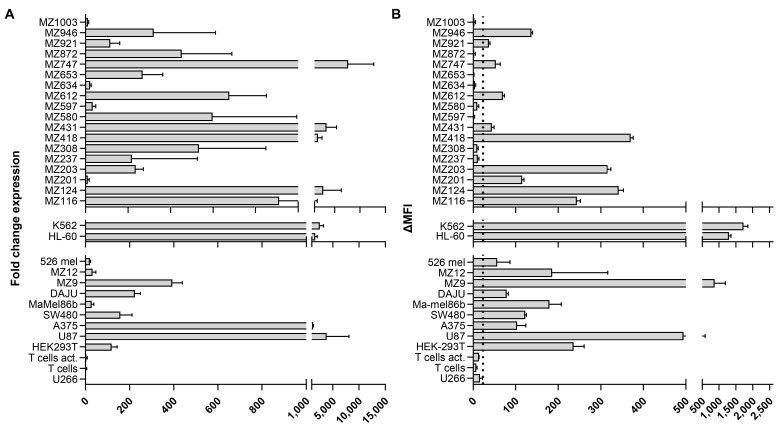
Quantification of B7H6 transcript and surface expression in AML and melanoma cell lines and primary AML blasts samples. B7H6 expression levels were quantified on the transcript and surface expression level for primary AML and melanoma samples, and established tumor cell lines. (**A**) B7H6 mRNA expression was examined by qPCR using total RNA samples, along with B7H6 specific primers, and normalized to β-actin expression with relative expression of B7H6, calculated to U266 as B7H6-negative tumor cell line using the ΔΔCt method. qPCR was performed in technical replicates (n = 3), with the data presented as mean fold-change relative to U266 and error bars indicating the SD. (**B**) B7H6 surface expression was assessed by flow cytometry using an anti-B7H6 mAb. Surface expression is represented as the difference in mean fluorescence intensity (ΔMFI) of the B7H6-directed antibody to isotype control, with error bars indicating the SD between technical replicates (n = 2). The dotted line defines ∆MFI levels equal or below the ∆MFI of U266 and was used as a cut-off to determine the borderline of ∆MFI for positive B7H6 staining since B7H6 expression on U266 cells was very weak and considered background. Fluorescence signals were acquired using a BD FACS Canto II flow cytometer.

To examine B7H6 expression on AML and melanoma as a representative solid tumor target for CAR T therapy, we screened a panel of eighteen patient-derived AML samples and three primary melanoma samples (MZ12, MZ9, Ma-mel86b) as well as melanoma cell lines (A375, 526 mel, DAJU) and additionally U87 (glioblastoma) and HEK 293T (epithelial carcinoma) by real-time RT-PCR and flow cytometry (Figure 2A,B). The myeloid cell lines K562 and HL-60 were included as reference for high B7H6 expression (Figure 2A,B), while U266 cells have been shown to be either negative or express barely detectable amounts of B7H6 (Figure 2A,B) [23], and were therefore used to determine a cutoff line for the difference in mean fluorescence intensity (ΔMFI) values considered to represent no distinct B7H6 expression.

RT-PCR analyses revealed B7H6 mRNA in ≥80% of all primary AML and in all patient-derived melanoma samples and melanoma cell lines tested (Figure 2A). Interestingly, in contrast to primary melanoma, we could not detect B7H6 cell surface expression in 5 out of 18 primary AML samples (28%) with detectable B7H6 mRNA (Figure 2B). Moreover, transcript levels were quite variable and did not correlate with the surface B7H6 antigen density (Figure 2A,B), indicating that additional post-transcriptional mechanisms might control expression. We further examined upregulation of B7H6 on activated T cells following polyclonal stimulation using anti-CD3 (OKT-3) and anti-CD28 mAbs in the presence of high dose IL-2 (500 u/mL) for three days. In contrast to recent findings [28], we did not detect B7H6 mRNA and cell surface expression above background levels (Figure 2A,B).

Collectively, our data suggest that B7H6 is widely expressed on AML and melanoma and therefore represents an interesting target for CAR T therapy. However, its expression appears variable due to post-transcriptional regulation.

### 2.3. NKp30 CAR/TCR^KO^ T Cells Are Effectively Stimulated with B7H6 Positive Tumor Targets

**Figure 3:**
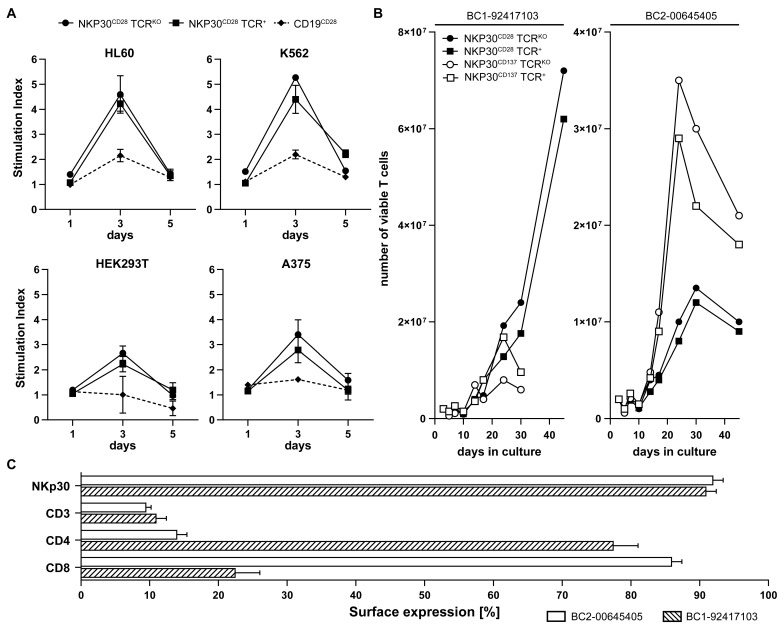
Assessment of proliferation and phenotypic characterization of CAR T cells upon antigen-specific stimulation tumor cell lines. (**A**) The proliferative capacity of generated NKp30-CD28 CAR TCR^+^ and NKp30–CD28 CAR TCR^KO^ T cells was assessed using ^3^H-thymidine proliferation assays. T cells from BC 1-92417103 were co-cultured at a 1:1 ratio with B7H6-positive tumor cell lines (HL-60, K562, HEK293T, and A375) for one, three, or five days, with ^3^H-thymidine added to quantify DNA synthesis. Cells were subsequently harvested, and radioactivity was quantified as counts per minute (cpm) and normalized to background proliferation of unstimulated T cells with values indicating the mean of technical replicates (n = 3) and SD. Anti-CD19-CD28 CAR TCR^KO^ T cells served as specificity control. (**B**) Proliferation of the NKp30-CD28 CAR TCR^+^ and NKp30-CD28 CAR TCR^KO^ or NKp30-CD137 CAR TCR^+^ and NKp30-CD137 CAR TCR^KO^ T cells generated from buffy coat BC 1-92417103 (NKp30-CD28 CAR T cells) or BC 2-0064505 (NKp30-CD137 CAR T cells) was assessed upon restimulation with B7H6^+^ HL-60 cells at different timepoints, represented as cell numbers. (**C**) Quantification of NKp30-CAR expression and immunophenotyping of the effector cells on day 10 post-transduction by flow cytometry analysis. Fluorescence signals were acquired using a BD FACS Canto II flow cytometer.

To examine NKp30 CAR-mediated antigen-specific proliferation depending on the B7H6 expression level, we co-cultured NKp30-CD28 CAR T cells with representative target cell lines, expressing high (K562, HL-60), intermediate (HEK293T) and low (A375) levels of B7H6 (Figure 2B). Both NKp30-CD28 CAR TCR^KO^ and NKp30-CD28 CAR TCR^+^ redirected T cells showed high proliferation upon recognition of K562 and HL60, and less but still solid responses to A375 and HEK293T targets reflecting B7H6 expression levels (Figure 3A). Response rates of NKp30-CD28 CAR TCR^KO^ effectors appeared to be slightly higher, indicating that CAR redirected T cells might confer more pronounced signaling in the absence of a competitive TCR/CD3 complex (Figure 3A). In contrast, anti-CD19-CD28 TCR^KO^ CAR T cells used as controls showed only low background reactivity, confirming that NKp30 CAR induced proliferation was specific and not alloantigen driven in the presence of residual endogenous TCR (Figure 3A).

Using PBMC isolated from buffy coats (BCs) of different donors as a source for T lymphocytes, we further compared the expansion kinetics of NKp30 CAR redirected T cells containing either CD28 or CD137 costimulatory domains. Both NKp30CD28 CAR TCR^KO^ and NKp30-CD137 CAR TCR^KO^, as well as NKp30-CD28 CAR TCR^+^ and NKp30-CD137 TCR^+^ T cells from different BCs (n = 4), here shown for the BC 1-92417103 (BC1) and BC 2-00645405 (BC2), were co-cultivated with irradiated HL60 targets at a 1:2 E/T ratio and T cell numbers determined after 5, 10, 17, 24, 30 and 45 days (d) of co-cultivation (Figure 3B). Flow cytometric analyses performed on day 14 of culture showed comparable NKp30 CAR (90%) and residual CD3 (15–17%) expression of TCR^+^ T lymphocytes in T cell cultures from both BCs. However, the ratio of CD4^+^ versus CD8^+^ T cells differed greatly between BC 1, consisting of 70% CD4^+^ and 30% CD8^+^ T lymphocytes, and BC 2, which contained only 15% CD4^+^ but 85% CD8^+^ T cells (Figure 3C). Additional staining for CD27 and CD45RA [29] revealed that the majority of cells were CD45RA^−^/CD27^−^ reflecting T effector memory cells (about 50%), followed by approximately 20% of CD45RA^+^CD27^−^ T effector cells and 10–12% CD45RA^+^/CD27^+^ naive T cells in both CD28 or CD137 costimulatory subsets of NKp30-CAR TCR^KO^ and NKp30-CAR TCR^+^ T cells The initial cell concentration of 2 × 10^6^ engineered T cells initially decreased but progressively recovered after electroporation, transduction, and puromycin selection throughout the first 10 days of culture until continuous and strong proliferation was observed (Figure 3B). NKp30-CD28 CAR T cell growth was more effective, yielding 7.5 × 10^7^ cells after 40 days as opposed to 3.5 × 10^7^ NKp30-CD137 CAR T cells. Moreover, exponential growth of NKp30-CD137 CAR T cells was faster compared to NKp30-CD28 CAR T cells and reached its maximum at 20–22d for NKp30-CD137 versus 40d for NKp30-CD28 CAR T cells (Figure 3B).

In line with previous work [30], costimulation through CD28 preferentially stimulated CD4 T^+^ cells whereas CD137-mediated costimulation promoted CD8^+^ T cell expansion (Figure 3C). Additionally, the observation that CAR-redirected TCR^KO^ T cells proliferated better than their TCR^+^ counterparts suggests that activation by CD3ζ signaling is likely more effective in TCR^KO^ CAR T cells due to less abundant competition of CD3 interaction with endogenous TCR (Figure 3B).

### 2.4. NKp30 CAR TCR^KO^ T Cells Produce High Amounts of IFN-γ upon Recognition of B7H6

**Figure 4:**
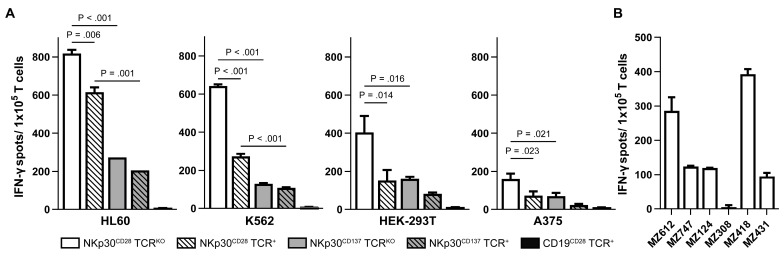
IFN-γ ELISpot analysis of NKp30-CAR T Cells. To evaluate the activation and cytokine production of generated NKp30-CD28 CAR TCR^KO^, NKp30-CD28 TCR^+^ and NKp30-CD137 CAR TCR^KO^ or NKp30-CD137 CAR TCR^+^ T cells, IFN-γ ELISpot analysis was performed with anti-CD19-CD28 CAR TCR^+^ T cells as specificity control. CAR T cells from BC 1 or BC 2 were co-cultured at an effector-target ratio of 2:1 with B7H6-positive tumor cell lines (**A**) HL-60, K562, HEK293T, and A375 or (**B**) primary AML blasts for 36 h. Specific cytokine production is shown as mean IFN-γ spots with error bars representing the SD of technical replicates (n = 3). Statistical analysis was performed using GraphPad prism (version 10.4) and Student’s *t*-test.

To examine NKp30-CD28 and NKp30-CD137 CAR T cells for effector functions, we first tested for their ability to release IFN-γ upon B7H6 recognition following coculture with HL60, K562, HEK293T, and A375 targets at a 2:1 E/T ratio. Prestimulated NKp30-CD28 CAR TCR^KO^ T cells elicited superior amounts of IFN-γ as compared to NKp30-CD137 CAR TCR^KO^ (Figure 4A), whereas anti-CD19-CD28 CAR TCR^KO^ T cells included as specificity control elicited no reactivity (Figure 4A). The NKp30-CD28 CAR TCR^+^ subset also demonstrated significant IFN-γ release but less compared to NKp30-CD28 CAR TCR^KO^ T cells as particularly observed upon stimulation with HLA deficient K562, again indicating that CAR-mediated T cell activation is more pronounced in the NKp30-CD28 CAR TCR^KO^ subset.

Accordingly, NKp30-CD28 CAR TCR^KO^ T cells released IFN-γ following co-incubation with several primary AML samples (Figure 4B), although amounts of IFN-γ produced did not always correlate with B7H6 expression levels detected on these AML samples as, e.g., seen for MZ124 (see Figure 2A). We therefore calculated a Pearson correlation coefficient for all primary AML samples depicted in Figure 2 and revealed no significant correlation of cytotoxic activity observed for IFN-γ in response to B7H6 mRNA transcript levels (r = −0.20, *p* = 0.669) or surface expression (r = 0.57, *p* = 0.238). However, we found a moderate positive correlation of B7H6 surface expression to IFN-γ release (r = 0.57) that might indicate a dependency, but more samples are needed to verify this trend.

### 2.5. NKp30 CAR TCR^KO^ T Cells Elicit Strong B7H6-Mediated Cytotoxicity In Vitro and in a NSG Melanoma Xenograft Model

In addition to cytokine release, we further evaluated the B7H6-mediated anti-tumoral cytotoxic response of NKp30 CAR engineered T lymphocytes in vitro and in vivo.

NKp30 CAR TCR^KO^ T cells and their TCR^+^ counterparts, generated from either BC 1 or BC 2 and used on d25 of expansion (see Figure 3B), were co-cultured with FLuc expressing tumor target cell lines HL-60, K562, HEK 293, and A375 for 20 h followed by measuring residual luciferase activity. Anti-CD19-CD28 CAR TCR^KO^ T cells served as specificity control.

**Figure 5:**
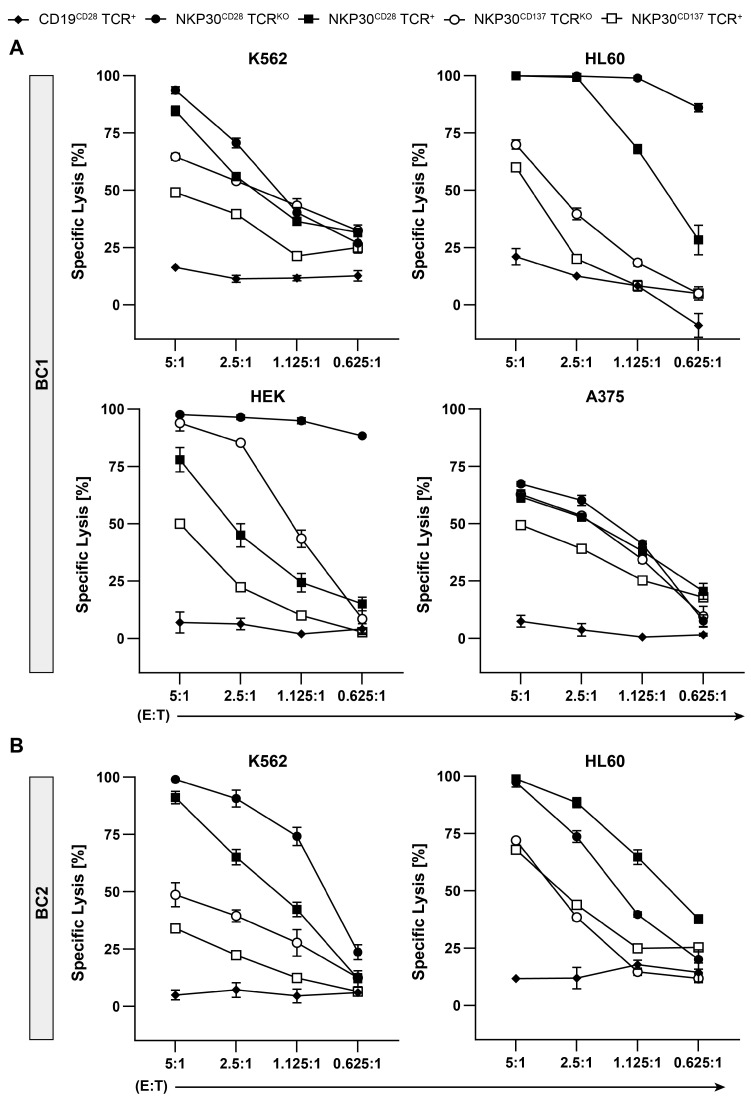
Cytolytic activity of NKp30-CAR T Cells. To evaluate the cytolytic activity of the different NKp30-CAR TCR^+^ and CAR TCR^KO^ T cells, bioluminescence-based FLuc cytotoxicity assays were performed. (**A**,**B**). NKp30 CAR redirected T cells of both subsets derived from two distinct PBMC donors (BC 1 and BC 2; see Figure 3A,B) were co-cultured with the FLuc expressing tumor target cell lines K562, HL60, HEK-293T, and A375 at the indicated E:T ratios for 16 h, with anti-CD19-CD28 CAR TCR^KO^ T cells as specificity control. Luminescent signal was measured and mean specific lysis and SD from technical replicates (n = 3) calculated as described in Material and Methods are depicted. One representative out of three experiments is shown.

In support of the IFN-γ data (see Figure 4), BC 1 derived NKp30-CD28 CAR TCR^KO^ T cells killed nearly 100% of K562, HL-60, and HEK as targets at a 5:1 E/T ratio (Figure 5A), whereas cytotoxicity elicited by NKp30-CD137 CAR TCR^KO^ T effectors was less consistent and ranged from 70 to 100% (Figure 5A). While cytotoxic responses by NKp30-CD28 CAR TCR^+^ T cells were similar to TCR^KO^ effectors, NKp30-CD137 CAR TCR^+^ T cells were clearly less effective in inducing cytotoxicity to all four targets at a 5:1 ratio (Figure 5A). Moreover, despite low B7H6 surface expression on A375 compared to the other three targets (see Figure 2B), both NKp30-CD28 CAR TCR^KO^ and NKp30-CD28 CAR TCR^+^ T cell subsets as well as NKp30-CD137 TCR^KO^ T lymphocytes elicited ≥70% cytotoxicity, indicating high avidity of NKp30 CAR TCR^KO^ T cells at limited B7H6 expression levels (Figure 5A).

Similar results were obtained when NKp30 CAR engineered T lymphocytes derived from BC 2 with higher numbers of CD8^+^ T cells were examined for B7H6-mediated cytolytic activity, suggesting that NKp30-CD28 CAR T effectors were more effective in vitro (Figure 5B). Since the amount of NKp30-CD137 CAR TCR^KO^ T cells was limited (see Figure 3B), cytolytic activity was only tested against K562 and HL60 targets (Figure 5B).

**Figure 6:**
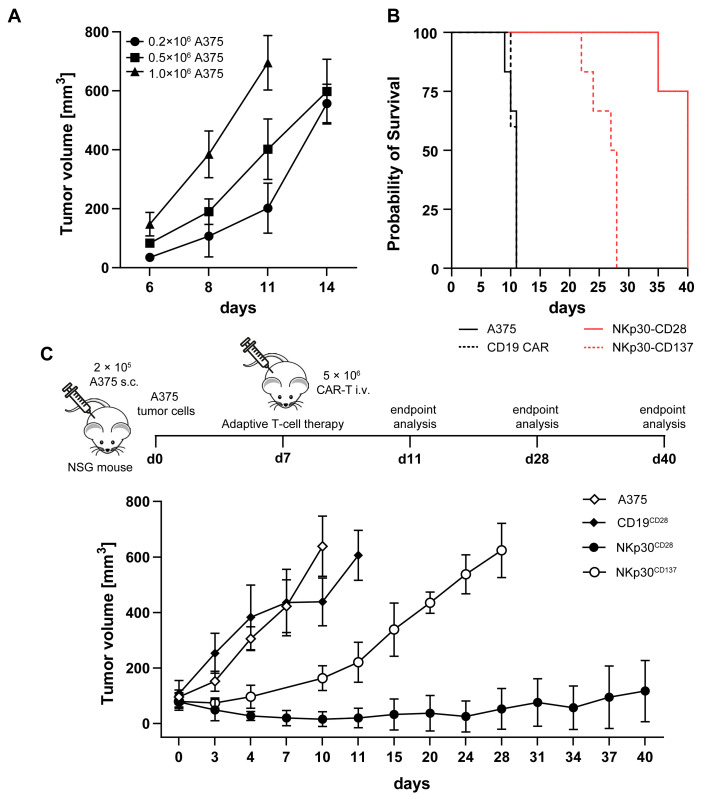
NKp30-CAR mediated induction of anti-tumor immunity to an A375 xenograft mouse model. (**A**) Tumor growth kinetics were established in NSG mice by subcutaneous injection of A375 cells into the left flank. A total number of 0.2, 0.5, or 1 × 10^6^ cells per mouse were used with the mean tumor volume and SD of replicates (n = 3) represented. For evaluation of the CAR-based therapeutic interventions, 0.2 × 10^6^ A375 were injected subcutaneously. Seven days post-engraftment, 5 × 10^6^ NKp30-CD28 CAR TCR^KO^ and NKp30-CD137 CAR TCR^KO^ T cells were administered intravenously, using untreated and anti-CD19-CD28 CAR TCR^KO^ T cells as controls. Mice were euthanized for endpoint analysis when tumors exceeded 750 mm^3^. CAR T cell-treated groups consisted of seven mice, while control groups (untreated and anti-CD19-CAR) included six mice. (**B**) Kaplan–Meier survival curves and (**C**) representative mean tumor volumes of two independent experiments are shown.

Next, we wanted to examine NKp30-CD28 CAR TCR^KO^ or NKp30-CD137 CAR TCR^KO^ T cells and their TCR^+^ counterparts in vivo ideally to primary AML expressing moderate to intermediate B7H6 levels to evaluate therapeutic efficacy under these conditions. However, as engraftment of AML blasts with appropriate B7H6 expression levels (MZ201, MZ946 and MZ116; Figure 2B) was not sufficiently robust and reproducible, we chose eGFP/FLuc expressing A375 melanoma cells to be engrafted in a NSG xenograft model. Initial tumor-dose kinetic studies revealed rapid tumor growth following subcutaneous injection into the left flank of NSG mice (Figure 6A). We therefore selected a tumor cell dose of 0.2 × 10^6^ to obtain a well palpable tumor volume of 100 mm^3^ on day seven as a time point to start CAR T therapy (Figure 6A).

Upon intravenous transfer of a single dose of either 5 × 10^6^ NKp30-CD28 TCR^KO^ or likewise 5 × 10^6^ NKp30-CD137 CAR TCR^KO^ T cells and anti-CD19-CD28 CAR TCR^KO^ T cells as specificity control supplemented with 1.000 IU IL-2/dose, tumor growth and overall survival of A375 tumor engrafted NSG recipients was determined (Figure 6B,C). When the tumor size reached a volume of 750 mm^3^, mice were killed for endpoint examination. Both untreated (A375 only, n = 6) or control-treated groups (anti-CD19 CAR T cells, n = 6) were terminated on day 10 and 11, respectively, to prevent tumor volumes >750 mm^3^ (Figure 6B). Whereas NSG tumor-bearing mice (n = 7) treated with NKp30-CD137 CAR TCR^KO^ T cells derived from BC 2 showed an initial anti-tumor response but progressed 10 days post T cell injection (Figure 6B,C), we observed a superior and long-lasting tumor immunity in the NKp30-CD28 CAR TCR^KO^ T cell-treated group (n = 7). Only one out of seven mice exhibited end-stage tumor burden on day 35 post CAR T therapy into A375 engrafted NSG recipients, while the remaining mice had stable disease with measurable tumor burden of around 150 mm^3^ on day 40 when the experiment was terminated (Figure 6B,C). These in vivo data demonstrate that NKp30-CD137 TCR^KO^ CAR T therapy led to a transient therapeutic anti-tumor effect. However, a single dose of NKp30-CD28 TCR^KO^ CAR T cells resulted in significant tumor regression and sustained stable disease for up to 40 days.

To examine tumor growth and metastasis in control and anti-CD19-CD28 CAR T-treated recipients, mice were analyzed for GFP expression of the A375 tumor cells within the tumor, spleen, and bone marrow (BM) by flow cytometry. (Figure S1). While 51% and 53% of GFP^+^ cells were found within tumors in both groups (Figure S1), we could detect only low amounts of GFP expressed in the spleen and BM. Interestingly, ex vivo analysis of tumor progression in one recipient following NKp30-CD28 TCR^KO^ CAR T-treatment revealed no measurable B7H6 cell surface expression in the isolated cells from the tumor specimen (Figure S2).

In conclusion, NSG mice exhibited specific and potent NKp30-CD28 TCR^KO^ CAR-mediated anti-tumor responses against A375-B7H6^+^ melanoma. While therapeutic efficacy with NKp30-CD137 TCR^KO^ CAR T cells was limited and not durable, adoptive transfer of NKp30-CD28 CAR TCR^KO^ T cells resulted in profound tumor regression.

## 3. Discussion

Despite ongoing research, the identification of a valuable target antigen with high tumor specificity, but ideally no or very low off-target effects, remains the main challenge for effective CAR T cell therapy in AML [4,6]. Since the B7 family member B7H6 has been reported to be expressed on a broad range of different solid tumors as well as hematological neoplasia [18,19,21,22,23] but is not found on hematopoietic cells of healthy individuals and rarely expressed on normal tissues [18,19,21,31,32], we studied the expression of B7H6 in patient-derived AML samples compared to established AML cell lines in detail, both at mRNA and cell surface expression level. In addition, we examined B7H6 expression on some primary melanoma samples and melanoma as well as other tumor cell lines, as B7H6 might also be an interesting target for CAR T therapy in melanoma.

Our B7H6 expression analyses revealed that in addition to the established leukemia, melanoma, and epithelial tumor cell lines tested, at least 40% of all primary AML blasts expressed B7H6 at the cell surface. Moreover, all patient-derived melanoma samples exhibited B7H6 antigen on the cell surface above a ΔMFI of 25 as determined by flow cytometry, and protein surface expression correlated with the amount of B7H6 transcripts quantified by RT qPCR. However, in some AML samples, such as MZ747, MZ580, and MZ431, transcript levels poorly correlated with cell surface expression of B7H6, suggesting post-transcriptional regulatory mechanism (s) or reduced surface expression by protease-mediated shedding [20,32]. Conversely, we found an inverse correlation of B7H6 mRNA levels and B7H6 cell surface expression on melanoma samples MZ12 and Ma-mel86b.

Various publications suggest that B7H6 expression is regulated by different mechanisms. Firstly, the ligand of the NCR NKp30 has been shown to be regulated at the transcriptional level by the proto-oncogene Myc [33]. As Myc expression is regulated, e.g., in myelodysplastic and leukemic stem cells by the signal transducer and activator of transcription 3 (STAT3) which becomes activated in response to IFN-γ and IL-6 [34,35], proinflammatory cytokines might promote expression of B7H6. Additionally, bromodomain-containing protein 4 (BRD4), a member of the BET family of epigenetic readers and overexpressed in AML cells, was found to bind to regulatory elements of the B7H6 promoter following histone acetylation to promote B7H6 transcription [24]. Secondly, B7H6 expression has been reported to be regulated by post-transcriptional mechanism (s) as, e.g., the small nuclear ribonucleoprotein (snRNP) polypeptide A (SNRPA) involved in the U1snRNP spliceosome was shown to upregulate B7H6 expression by promoting B7H6 pre-mRNA maturation in hepatocellular carcinoma [36]. Furthermore, de novo expression of B7H6 on the cell surface as a stress-induced response protein was demonstrated to result from perturbations of endoplasmic reticulum (ER) homeostasis and ER-stress leading to an unfolded protein response [20] commonly observed in cancer [37]. Finally, B7H6 expression has been found to be post-transcriptionally regulated by disintegrin and metalloproteinase-mediated shedding of the B7H6 ectodomain, resulting in escape from NK cell-mediated recognition [38]. As we did not follow up with the loss of B7H6 expression on melanoma cells isolated ex vivo from a relapsed NSG mouse treated with NKp30-CD28 CAR TCR^KO^ T therapy, we can only speculate that the appearance of a B7H6 negative melanoma clone was possibly due to dysfunctional transcriptional/post-transcriptional control mechanism described or metalloproteinase-mediated shedding.

Of note, we observed complete loss of B7H6 surface expression upon transfection of B7H6 cDNA that lacks the intracytoplasmic region, suggesting that signaling motifs within the intracellular region such as the reported SRC homology 2 and 3 domains might also play a role in regulating surface expression (unpublished data) [18]. In addition to tumors, expression of B7H6 has been shown to be upregulated under inflammatory conditions in proinflammatory monocytes and neutrophils [39] and thus might be induced by NKp30 CAR T cells expressing, e.g., IFN-γ and TNF-α.

Collectively, our data demonstrate that B7H6 is not only upregulated on tumor cell lines of different origins as previously shown but is also expressed on a substantial number of primary AML and melanoma samples. The differences observed in some AML samples showing an inconsistent correlation of RNA and B7H6 cell surface expression (see Figure 2) might at least partially be explained by the different transcriptional and post-transcriptional mechanisms described above and would need further research to address these mechanisms in more detail.

To examine B7H6 as a potential target for CAR T therapy to AML and melanoma, we thus developed two different second-generation CAR constructs composed of the extracellular domain of the NCR NKp30 and either CD28 or CD137 as costimulatory domains fused to the CD3ζ signaling moiety. As the efficacy of CAR redirected autologous T cell products depends on the intrinsic fitness of patient-derived T lymphocytes and comes at the cost of personalized production and treatment, we used allogeneic T cells and applied genomic CRISPR/Cas9 editing to delete endogenous TCR expression followed by retroviral NKp30-CAR gene transfer. This approach was previously reported for highly efficient multiplex genome-edited CAR T cell generation [40] and abolishes the risk of inducing immunogenicity to a protein of bacterial origin due to transient activity of the CRISPR/Cas9 sgRNA complex [41]. Moreover, CRISPR-engineered CAR19 universal T cells have been recently evaluated in a phase I clinical trial for the treatment of children with refractory B-cell leukemia [42].

Using different BCs from healthy donors as a source of T lymphocytes revealed substantial differences in the B7H6 antigen-driven expansion of NKp30-CD28 TCR^KO^ versus NKp30-CD137 CAR TCR^KO^ redirected T cells. Thus, confirming previously published data [30], T cells expressing the NKp30-CD28 CAR resulted in predominant outgrowth of CD4 positive T cells, whereas the majority of NKp30-CD137 CAR T cells were of CD8 positive subtype. This suggests that, in addition to the initial CD4/CD8 ratio of T cells present in a given donor lymphocyte product, the costimulatory domain used for CAR T cell engineering will likely result in a bias of CAR redirected T cells either to the CD4 or CD8 subset. We further observed continuous expansion and a two-fold higher concentration of T cells expressing the NKp30-CD28 CAR after 40 days of culture in contrast to NKp30-CD137 CAR redirected T cells, reaching a maximal cell number after 20 days of culture and then started to decline. Moreover, since our NKp30-CD28 CAR consists of a CD28 TMD and ICD, this might result in higher secretion of IL2 and sustained proliferation compared to the NKp30-CD137 CAR containing a CD4 TMD as described [43]. CD28 TMD-containing CARs can recruit and dimerize with endogenous CD28, which normally exists as a homodimer on the cell surface, via a four amino acid motif in the TMD [44], leading to phosphorylation of endogenous CD28 and stronger signal transduction, facilitating CAR T cell activation in the context of low levels of CAR antigen [45]. Finally, as CAR redirected T cells with residual TCRαβ expression expanded less and were apparently not alloantigen driven by HL60 targets expressing non-matched HLA class I and class II alleles, this might indicate that competitive co-expression of the residual endogenous TCR-CD3 complex and the CAR-CD3ζ moiety affects the outcome of CD3 signaling.

In addition to prolonged proliferation and higher cell yield, NKp30-CD28 CAR TCR^KO^ T cells elicited superior IFN-γ release upon recognition of B7H6 expressed on leukemia and melanoma cell lines and primary AML blasts. Accordingly, strong cytotoxic responses by NKp30-CD28 CAR TCR^KO^ T cells to tumor cell lines representing AML and melanoma, not only in the presence of high B7H6 expression (HL60, K562) but also when B7H6 expression was intermediate (HEK-293T) or low (A375), were observed in vitro, which is in line with the previous finding that CAR T cell activation is facilitated by CD28 containing CARs in the presence of low antigen [45]. In order to evaluate the efficacy of CAR engineered T cells in vivo, we ideally sought to test NKp30 CAR redirected T cells in a patient-tailored NSG AML-xenograft model expressing moderate B7H6 levels on the AML cells. However, as shown previously, primary AML samples do not reproducibly engraft and home to murine bone marrow with consistent efficacy [46]. Hence, we were not successful in establishing an AML-xenograft model using available AML samples with intermediate B7H6 expression. We therefore compared the therapeutic efficacy of NKp30-CD28 and NKp30-CD137 CAR TCR^KO^ T cells in vivo in an A375-melanoma NSG xenograft model with B7H6 expression levels similar to, e.g., AML samples MZ116, MZ201 and MZ612. NKp30-CD28 TCR^KO^ CAR T lymphocytes elicited a robust anti-tumor response and sustained control of tumor burden, whereas NKp30-CD137/TCR^KO^ CAR T cells demonstrated partial and transient anti-tumor reactivity. These data confirm previously and very recently published results showing that CD28-containing CARs confer greater functionality [47,48,49] and CD4^+^ T cells expressing CD28 CAR constructs can control large, established tumor xenografts [50]. However, despite some delay in tumor progression seen upon transfer of NKp30-CD137 CAR TCR^KO^ T cells, we could not observe better persistence of NKp30-CD137 CAR TCR^KO^ engineered T cells compared to CD28-costimulated CAR T lymphocytes [47,48,51]. This might at least partially be explained by the fact that the infused NKp30-CD137 CAR TCR^KO^ T cells were mainly of the CD8 subtype known to produce less IL-2 compared to CD4^+^ T cells, and NSG mice do not provide any IL-2 or IL-15 to support CD8 T cell homeostasis. Additionally, CD28 signaling, as compared to 4-1BB, has been shown to result in faster and larger magnitude changes in protein phosphorylation, influencing the response and differentiation of effector T cells [51]. Nevertheless, it might also be conceivable that NKp30 CAR-mediated T cell immunity in an AML xenograft model could reveal some differences in CAR T cell reactivity due to, e.g., different location of the tumor. We therefore envisage to evaluate CAR T cells in an AML xenograft model upon availability of suitable AML samples.

As upregulation of B7H6 predominantly on CD8^+^ T cells has recently been reported [28], we further tested for expression of B7H6 on anti-CD3/anti-CD28 mAb stimulated T cells and on NKp30 CAR T cells. However, in contrast to the data reported by Kilian et al., we could not detect B7H6 expression above background and peak levels 72 h post activation in polyclonally primed T cells. Moreover, in our experiments, mostly CD4 expressing NKp30-CD28 CAR T cells continuously expanded until day 40 while expansion of primarily CD8 positive NKp30 CD137 CAR T cells started to decline after three weeks of expansion. Although we cannot fully exclude fratricide or autoreactivity of preferentially NKp30-CD137 CD8^+^ CAR T cells during expansion and in our functional in vitro and in vivo studies, we rather propose that this decline was primarily due to mechanisms like IL-2 mediated activation-induced cell death by IL-2 supplementation in a primary CD8^+^ T cell subset or functional exhaustion. The fact that expanded NKp30 CD137 CAR TCR^KO^ T cells, which could not elicit TCR-mediated fraticide, declined as well, supports this assumption. Finally, we did not detect NK cells in our T cell expansion cultures to elicit NK-cell mediated T cell killing via NKp30. However, the potential mechanism (s) associated with the reduction in NKp30 CD137 CAR T cells after initial expansion need to be further elucidated.

Since, in addition to B7H6, the Bcl-2 associated athanogene 6 (BAG6, synonym Bat3) binds to the NCR NKp30, NKp30 CAR redirected T cells could also be stimulated following recognition of BAG6. However, as in contrast to B7H6, this ligand has been found to be primarily secreted and expressed on extracellular vesicles (EV) [52], and as to our knowledge, there is no commercial mAb available for staining; we did not further examine expression of BAG 6 in our study. However, we did not observe any putative inhibitory effects of EV-BAG6 bound to the NK30 CAR on our redirected T cells.

Taken together, our studies complement and extend previous reports on the preclinical evaluation of NKp30 CAR T therapy to B7H6-expressing tumors [26,27,53,54] in immunocompetent mice using B7H6 transfectants, since B7H6 is a pseudogene in mice [26,27]. This might promote additional endogenous anti-tumoral immunity by murine T cells not tolerized to human B7H6. The data published by Hua et al. and Gacerez et al. report the development of fully humanized scFvs targeting high B7H6-expressing tumors and thus focus on scFvs for anti-B7H6 CARs that potentially spare non-tumor cells expressing low levels of B7H6 [53,54]. However, our data indicate that NKp30-based CAR T cells can detect low to intermediate B7H6 expression, which, given our observations on differential B7H6 expression levels in AML, might facilitate anti-tumor reactivity not only to high B7H6-expressing tumors.

In conclusion, our studies show that B7H6 is not only commonly expressed on established tumor cell lines of different origins but also on primary AML and melanoma samples, which further highlights the potential of the de novo expressed tumor-antigen B7H6 for CAR T therapy. Moreover, we show that allogeneic NKp30-CD28 CAR TCR^KO^ T cells induce potent anti-tumor immunity to tumors expressing not only high but also intermediate and low levels of B7H6, including AML and melanoma.

## 4. Materials and Methods

### 4.1. Blood Samples, Primary Cells, and Cell Lines

Buffy coats were provided by the Transfusion Blood Center of the University Medical Center Mainz (UMC) following the Declaration of Helsinki. Written consent provided by healthy volunteers before collecting whole blood samples was approved by the Transfusion Blood Center of the UMC. Primary AML samples were obtained from patients after informed consent according to a study protocol (No. 837.185.00 (2551)) approved by the Ethics Committee of the Landesaerztekammer Rheinland-Pfalz. Peripheral blood mononuclear cells (PBMCs) were isolated by Ficoll density gradient centrifugation (Sigma-Aldrich, Steinheim, Germany) and cultured using AIM-V medium (Life Technologies-Thermo Fisher Scientific, Darmstadt, Germany) supplemented with 10% human serum (HS). For polyclonal T cell stimulation, PBMCs were subjected to anti-CD3/CD28 monoclonal antibodies (mAb), and T cells were immunomagnetically isolated and stimulated using expander beads (Miltenyi Biotec, Bergisch Gladbach, Germany). Primary AML cells were cultured in RPMI-1640 medium (Life Technologies) supplemented with 10% HS and 1% Penicillin/Streptomycin (P/S) (Sigma-Aldrich). The retroviral packaging cell line Phoenix-Ampho and target cell lines, including A375 and HEK-293T, were cultured in DMEM (Life Technologies), whereas HL-60, and K562 were cultured in RPMI-1640 medium supplemented with 10% FBS and 1% P/S.

### 4.2. Generation of Luciferase-Expressing Target Cells

To generate stable cell lines expressing firefly luciferase (FLuc)–eGFP fusion protein, K562, A375, HL60, and HEK-293T cells were transduced with a modified lentiviral vector (pLenti-EF1a-pac-T2A-eGFP/FLuc) kindly provided by Dr. C. Wölfel, III. Department of Medicine, UMC. Lentiviral particles carrying transfer vector were produced by transfecting HEK-293T cells with pLenti transfer vector, along with psPAX2 and pMD2.G, as helper plasmids using TransIT-LT1 transfection reagent (Mirus, Madison, WI, USA). Viral supernatants were harvested after 36–48 h, resuspended in RPMI-1640 (Life Technologies) containing 8 µg/mL polybrene (Sigma-Aldrich), and added to target cells.

### 4.3. Generation of NKp30-CAR TCR^KO^ T Cells

NKp30-based CAR constructs were generated by cloning sequences encoding the Lκ leader, ectodomain of NKp30, and the CD28/CD28 or CD4/CD137 (4-1BB) transmembrane domain (TMD) and costimulatory intracellular domain (ICD), along with CD3ζ signaling domain into a pMXs-IRES-puro vector using seamless NEBbuilder assembly. To obtain TCR α-chain knockout (KO) T cells, equal molar amounts of TCRα crRNA and tracrRNA (100 µM) were combined in IDT duplex buffer to anneal RNA complexes at 95 °C following cooling to RT. For transfection, 0.5 × 10^6^ activated T cells were electroporated with 1.5 µM Alt-RTM S.p. Cas9 3NLS, 1.8 µM guide RNA complex, and 1.8 µM Alt-RTM Cas9 electroporation enhancer using the Neon Transfection System (Thermo Fisher Scientific, Waltham, MA, USA) in 10 mL of buffer T and using three 1600 V, 10 ms pulses followed by incubation in AIM-V medium, supplemented with 10% HS and 600 U/mL interleukin-2 (IL-2) for 4 h. Retroviral gene transfer of the NKp30-CAR constructs was performed using 5 µg of each of the helper plasmids pCOLT-GALV and pHIT60 along with 10 µg of the transfer vectors. CAR T cells were expanded by weekly antigen-specific restimulation using irradiated B7H6^+^ HL-60 target cells.

### 4.4. Antigen-Specific Stimulation and Proliferation of CAR T Cells

Irradiated (700 Gy) B7H6-expressing HL60 cells (5 × 10^5^) were co-incubated with 1 × 10^6^ CAR T cells in AIM-V medium, 10% HS, and 200 IU IL-2/mL. T cell expansion was determined weekly after restimulation for up to 50 days.

### 4.5. Real-Time Quantitative PCR (qRT-PCR)

Total RNA samples were extracted from cultured cells using the NucleoSpin RNA Plus Kit (Macherey-Nagel, Düren, Germany) followed by reverse transcription to cDNA using the SuperScript III (Thermo Fisher Scientific), both according to the manufacturer’s instructions. Gene expression of B7H6 was examined by qPCR using PowerTrack SYBR Green (Applied Biosystems, Thermo Fisher Scientific, Darmstadt, Germany) and normalized to β-actin expression, with relative expression of B7H6 calculated to U266 as a B7H6-negative tumor cell line using the 2^−ΔΔCt^ method.

### 4.6. Flow Cytometry Analysis

For analysis of surface marker expression on tumor cell lines, primary AML blast on effector cell lines and flow cytometry was performed. Cells were stained using fluorophore-conjugated monoclonal antibodies against B7H6 (clone 875001, R&D, Minneapolis, MN, USA), CD3, CD4, CD8, CD27, CD45RA (BD Pharmingen, San Diego, CA, USA) or CD337 (NKp30) (BioLegend, San Diego, CA, USA), in addition to isotype control samples and fluorescent signal obtained using a FACSCanto II (BD Biosciences, Franklin Lakes, NJ, USA) cytometer and FlowJo (version 10.6.1) (Ashland, OR, USA) software.

### 4.7. Measuring IFN-γ Production by T Cells

To detect and quantify IFN-γ release of the transduced T cells, cells were co-cultured with different B7H6 targets overnight, followed by an IFN-γ ELISpot assay performed as previously described [55].

### 4.8. Bioluminescence-Imaging-Based Cytotoxicity Assay

To assess cellular cytotoxicity of CAR T cells, 2 × 10^4^ FLuc-expressing tumor cell lines were co-cultured at different effector-to-target (E:T) ratios at 37 °C for 20 h in the presence of 0.15 mg/mL D-luciferin in technical replicates (n = 3). Baseline luminescent signal was obtained by incubating target cells with culture medium (spontaneous cell death) or 1% Triton X-100 (maximum lysis) to normalize and quantify cell lysis. Luminescent signals were detected using a FLUOstar Omega ELISA reader.

### 4.9. Treatment of Tumor-Bearing Mice with NKp30 CAR T Cells

In accordance with national and institutional guidelines for animal care, 8 to 12-week-old NOD.Cg-Prkdc^scid^IL2R^tm1Wjl^/SzJ (NSG) mice were obtained from the Translational Animal Research Center of the Johannes Gutenberg University Mainz, Germany, to be used for in vivo experiments. Mice were injected subcutaneously with 0.2 × 10^6^ A375 cells, followed by a single injection of 5 × 10^6^ of NKp30 TCR^KO^ CAR T cells seven days later, when the tumor volume reached about 100 mm^3^. Mice were randomized into four cohorts, receiving either CD19-CAR/TCR^+^, NKp30-CD28 CAR, or NKp30-CD137 CAR T cells, with an additional untreated control group. Mice were sacrificed by cervical dislocation at a tumor volume of 750 mm^3^. Endpoint analyses included measurement of tumor volume and B7H6 surface expression on tumor cells isolated ex vivo from the primary tumor, spleen, and bone marrow.

### 4.10. Statistical Analysis

Student’s *t*-test or ANOVA were used to analyze differences between groups. Kaplan–Meier survival curves were used to analyze survival benefit. Statistical analysis was performed using GraphPad Prism (version 10.4.1) (Boston, MA, USA).

## Data Availability

The data are available from the corresponding author upon reasonable request.

## Supplementary Materials

The following supporting information can be downloaded at: https://www.mdpi.com/article/10.3390/ijms26178235/s1.
