# Supplementary material for: CRISPR/Cas9 TCR-Edited NKp30 CAR T Cells Exhibit Superior Anti-Tumor Immunity to B7H6-Expressing Leukemia and Melanoma
Source: Int J Mol Sci. 2025 Aug 25;26(17):8235. doi: 10.3390/ijms26178235 (PMC12428420; doi:10.3390/ijms26178235)
Supplement: Supplementary file 1 [file ijms-26-08235-s001.zip › ijms-3722185-supplementary.pdf]

Supplementary Material

CRISPR/Cas9 TCR-edited NKp30 CAR T cells exhibit superior anti-tumor immunity to B7H6-expressing leukemia and melanoma

Sedigheh Givi[1,6], Benedikt J. Lohnes[1,6], Saber Ebrahimi[1,6], Sophie Riedel[1,6], Sneha Khokhali[1,6], Shamsul A. Khan[1,6], Maximilian Keller[1,6], Catherine Wölfel[1,6], Hakim Echchannaoui[1,6,7], Ernesto Bockamp[5,6], Maya C. Andre[2,3], Hinrich Abken[4], Matthias Theobald[1,6,7], Udo F. Hartwig[1,6,7]

[1] Dept. of Medicine – Hematology & Medical Oncology, University Medical Center of the Johannes Gutenberg-University, Mainz, Germany
[2] Dept. of Hematology/Oncology and General Pediatrics, Children's University Hospital, University of Tübingen, Tübingen, Germany
[3] Dept. of Pediatric Intensive Care, University Children's Hospital, Basel, Switzerland.
[4] Leibniz Institute for Immunotherapy, Div. Genetic Immunotherapy, Regensburg, Germany
[5] Institute for Translational Immunology, University Medical Center of Johannes Gutenberg-University, Mainz, Germany
[6] Research Center for Immunotherapy, University Medical Center (UMC) of Johannes Gutenberg-University, Mainz, Germany
[7] German Consortium for Translational Cancer Research (DKTK), Partner Site Frankfurt/Mainz, Mainz, Germany

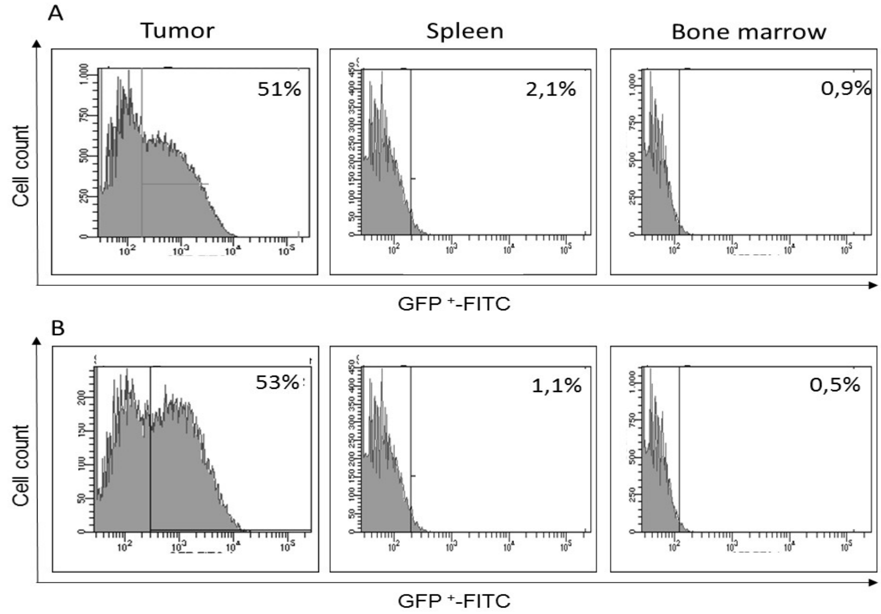

Fig. S1: Phenotypical analysis of GFP^+ tumor cells isolated from primary tumor site, spleen and bone marrow from NSG mice.
Following preparation of single cell suspension of ex vivo isolated tissue specimens GFP expressing tumor cells were detected in A) untreated mice and B) mice treated with NKp30-CD28 CAR TCR^{KO} T cells. Representative flow cytometric images from one experiment are shown.

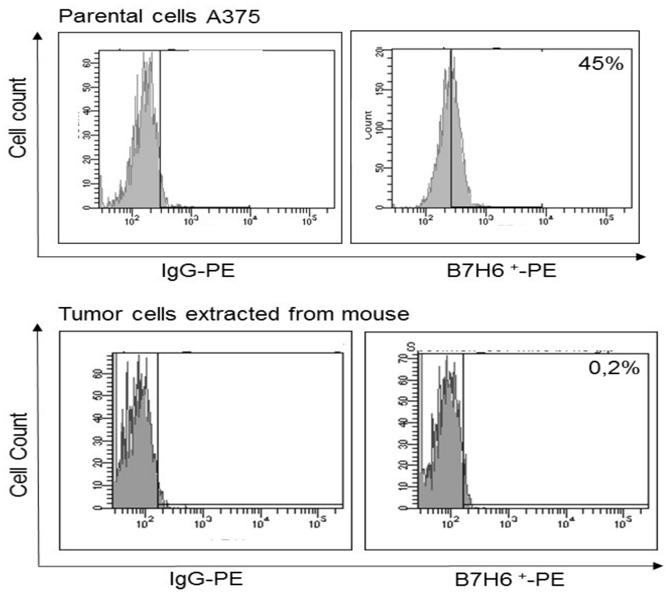

Fig. S2: The B7H6 expression of tumor cells isolated ex vivo from an A375 melanoma relapsed NKp30-CD28 CAR TCR^{KO} T-therapy treated NSG mouse. Following ex vivo isolation tumor cells were stained for B7H6 surface expression using the B7H6 and mouse IgG-PE antibody. Representative images of flow cytometry from one experiment are shown.
